# Ethical Reflections on Co‑Authoring With People With Lived Experience Across Health, Education and Community Settings

**DOI:** 10.1111/hex.70913

**Published:** 2026-10-29

**Authors:** Hilary Moss, Katie Fitzpatrick, Patricia O'Shea, Hannah Healy, Anne MacFarlane

**Affiliations:** ^1^ Health Research Institute University of Limerick Limerick Ireland; ^2^ Pain Excellence Centre Croom Orthopaedic Hospital HSE Mid West Limerick Ireland

**Keywords:** collaborative learning, ethics consultation, music, participatory action research, participatory decision making, participatory design, value (ethics)

## Abstract

**Introduction:**

Co‑authoring with people who have lived experience is increasingly recognised as essential to meaningful patient and public involvement (PPI) (Involve 2012). Yet genuine power‑sharing in academic writing remains rare, particularly in music and health research. Traditional research models position participants as ‘informants’, while academic authors retain interpretive authority. Participatory Learning and Action (PLA) offers principles and methods to shift research from extractive, top‐down models towards collaborative knowledge creation.

**Objective and Methods:**

This viewpoint argues that co‑authoring with lived‑experience contributors is not merely a methodological choice but an ethical stance requiring time, humility and structural disruption. Drawing on the experience of co‑editing a book with 11 lived‑experience authors, this article briefly examines the ethical, relational and practical challenges of co‑authorship through the lens of PLA reversals.

**Results:**

Nine themes are presented and discussed based on the authors reflections of co‐authoring a book with participants with lived experience.

**Conclusion:**

Co‐authoring with participants with lived experience is not only feasible but vital. PLA reversals support meaningful, ethical and impactful collaboration.

## 1 Introduction

Co‐authoring with people who have lived experience is increasingly recognised as essential to meaningful patient and public involvement (PPI) [1]. Yet genuine power‐sharing in academic writing remains rare, particularly in music and health research. Traditional research models position participants as ‘informants’, while academic authors retain interpretive authority. Participatory Learning and Action (PLA) offers principles and methods to shift research from extractive, top‐down models towards collaborative knowledge creation [2].

This viewpoint argues that co‐authoring with lived‐experience contributors is not merely a methodological choice but an ethical stance requiring time, humility and structural disruption. Drawing on the experience of co‐editing a book with 11 lived‐experience authors, this article briefly examines the ethical, relational and practical challenges of co‐authorship through the lens of PLA reversals [3].

## 2 Background: Co‐Authorship and Participatory Approaches

Although PPI is widely promoted, co‐authorship remains uncommon. Sarna‐Wojcicki and colleagues found that only 6% of participatory research articles positioned non‐academic collaborators as co‐authors, with most relegating contributors to acknowledgements [4]. In music‐and‐health literature, co‐authored accounts exist but rarely use explicit participatory frameworks. The discourse on health service delivery acknowledges the desirability of engaging with people with lived experience in a participatory manner—as key partners in the delivery and design of services [5]. However, research agendas and direction are often set by seasoned academics without true collaboration with people with lived experience.

## 3 Participatory Learning and Action (PLA)

PLA reversals (see Table 1) offer a structured way to interrogate power dynamics. They encourage researchers to shift from top‐down control to bottom‐up collaboration, from assuming knowledge to co‐developing knowledge and from dominating to empowering. This viewpoint uses these reversals to examine nine ethical themes that emerged during the co‐editing process.

**Table 1:** PLA reversals [5].

Reversals from….	To….
Speaking	Listening and hearing
Assuming knowledge	Sharing complex ‘knowledges’
Dominating	Empowering
Verbal	Visual and musical^a^
Top‐down	Bottom‐up and sideways in
Participants seen solely as beneficiaries	Participants seen as partners, collaborators and beneficiaries
Service provision conceived as ‘supply‐push’ from service providers towards service‐users, with little or no service user involvement	Service provision conceived as ‘demand‐pull’ from service‐users with extensive service user involvement in service design and delivery
Written and spoken word dominance^b^	Acceptance of creative and artistic forms of knowledge such as art, poetry, music and theatre

a The importance of musically inspired knowledge was important in our project, hence the addition of audio knowledge here.

b The use of audible and music‐based knowledge was an important addition in our work. We therefore propose one additional item to O'Reilly‐de Brún and de Brún's PLA reversals.

## 4 Ethical Themes in Co‐Authoring

### 4.1 Ownership, Rights and Power Reversal

Co‐authoring requires a deliberate redistribution of power. In this project, lived‐experience authors were positioned as first authors, and decisions about publication rested with them. Most chapters were co‐written, but the senior researcher was always secondary to the co‐author with lived experience, who was first author.

The prison‐based chapter revealed the limits of power reversal. Institutional control over writing materials, access and content review meant that power was never neutral or absent. Even with strong intentions towards equity, structural constraints shaped what could be written.

**Recommendation:** Prioritise lived‐experience authorship and decision‐making throughout the process.

### 4.2 Language and Naming

Language shapes representation and belonging. The editors (one of whom is an expert by experience) rejected labels such as ‘patient’ and debated alternatives before choosing to refer to contributors simply as people. The commitment to co‐developing knowledge rather than imposing categories was embedded from the earliest stages of the project, including recognition of preferred labels by experts by experience throughout the book. This reframing required deep reflection and dialogue.

**Recommendation:** Treat language as an ethical choice; interrogate labels that reinforce hierarchy. Language is never neutral—it is relational, contextual and deeply intertwined with identity, agency and power.

### 4.3 Supporting Writing Capacity and Time

Many contributors had never written a chapter before. Academic or practice‐based co‐authors played crucial roles in editing and co‐crafting narratives. One professional withdrew early, stating they ‘didn't have time’ for the careful work required—highlighting the tension between rhetorical inclusion and actual labour. In prison and in one healthcare facility, limited access to the co‐author slowed collaboration and raised concerns about whether agreement with edits reflected confidence or deference. Editors needed to be sensitive when suggesting edits, and remain true, wherever possible, to the original voice of the contributor.

**Recommendation:** Have honest conversations from the start about the time and effort involved in co‐authoring. Ensure adequate time and support for authors; avoid tokenistic inclusion.

### 4.4 Editorial Care and Voice Preservation

Editing required balancing clarity with authenticity. Editors returned tracked changes with messages emphasising that all edits were optional. Chapters were structured to foreground lived‐experience narratives, with professional reflections placed later. This approach aligns with PLA reversals that value listening and diverse ways of knowing.

**Recommendation:** Use transparent editorial processes that preserve voice and foreground lived experience.

### 4.5 Iterative Consent and Withdrawal

Consent was treated as ongoing. Some contributors reconsidered participation due to legal or family concerns. In two cases, edits were made after chapters were complete due to concerns of legal or family fallout from reading their experiences. Contributors were gifting their story to the world, at a cost of exposing their vulnerability and in prison, drafts required institutional approval, and the co‐author was mindful of potential consequences.

**Recommendation:** Maintain flexible, iterative consent processes; allow withdrawal at any stage.

### 4.6 Changes in Status and Loss of Stories

Illness, institutional barriers, transfers and death affected several chapters. One contributor was delayed in signing off edits due to restricted prison access; another's writing was delayed by sudden health deterioration. Researchers must be prepared to ‘lose a good story’ when circumstances prevent completion. Accepting uncertainty and prioritising contributor wellbeing over publication timelines was a necessary ethical condition of engaging in this work. Publication can only go ahead if, and when, the person with lived experience decides it is ready and can be withdrawn at any time. In proposing chapters, careful consideration was given to ensuring that the co‐author's participation was genuinely voluntary.

**Recommendation:** Consider the time delays, ethical considerations and participant wellbeing throughout the process of publication. We recommend asking the question: Am I *really* prepared to adhere to PLA approaches at the expense of my own publication and promotion?

### 4.7 Recruitment, Inclusion and Bias

Open calls for contributors proved less effective than inviting authors through professional relationships. Support was needed to encourage writers to share their story and to have the confidence to write. These approaches produced rich, meaningful co‐authored narratives but may have introduced bias (geographical and reduced breadth of health experiences represented).

**Recommendation:** Involve lived‐experience contributors in early conceptualisation and recruitment.

### 4.8 Invisibility, Anonymity and Posthumous Authorship

Some contributors could not be publicly named; others had died since agreeing to share their stories. Ethical clearance was revisited, and families were consulted. In carceral settings, anonymity risked displacing authorship onto academic partners. In another case, the death of the participant meant that family needed to be carefully consulted.

**Recommendation:** Attend meticulously to anonymity, ownership and recognition and note the time and energy involved in ongoing rigorous adherence to the highest ethical standards.

### 4.9 Sustained Relational Commitment and Safeguarding

Co‐authorship requires ongoing communication, flexibility and emotional safety. Safeguarding is essential to prevent distress or re‐traumatisation. Post‐publication duty of care emerged as a critical issue: once a story is published, it cannot be retracted.

**Recommendation:** Provide post‐publication support and anticipate long‐term impacts on contributors.

## 5 Discussion

Co‐authoring with experts by experience requires care, commitment, time and patience. Tokenism is quicker and less complicated. This viewpoint aligns with Devlin et al.'s six considerations for co‐authored research, including dual relationships; engaging with the ethics review board; roles, responsibilities, and safeguards and authorship.

Editorial responsibility also involves a degree of stewardship to ensure quality, consent, tone, respect, accuracy and the avoidance of sensationalism. However, this must be balanced carefully so that editorial oversight does not reinforce hierarchies that privilege professional over experiential knowledge. Transparent criteria for authorship and inclusion can help prevent this. Editors should continually question their own decision‐making—asking whether choices are motivated by safety, convenience or control—and strive for co‐ownership of both process and product.

Contributors' time, knowledge and emotional labour should be acknowledged at every opportunity. While the current structures of academic publishing do not provide financial remuneration for chapter authors, fair recognition must remain a priority. Authorship credit, inclusion in related outputs and events, and the provision of a personal copy of the book are small but meaningful gestures. Sustained collaboration demands continuous reflection on power and privilege, ensuring that professional partners remain accountable and responsive to the needs of their co‐authors.

## 6 Conclusion

Co‐authoring with people with lived experience is both feasible and vital. PLA reversals—listening, sharing knowledge, valuing diverse ways of knowing, bottom‐up design, empowerment and partnership—support ethical, meaningful and impactful collaboration.

Co‐authorship disrupts power structures that allow academics to speak for others. Those who are the subject of research should be co‐steering the process. Ethical co‐authorship is not an enhancement—it is a necessary transformation.

## Author Contributions

**Hilary Moss:** conceptualisation, investigation, writing – original draft, methodology, writing – review and editing, project administration. **Katie Fitzpatrick:** conceptualisation, investigation, writing – review and editing, methodology, validation, formal analysis. **Patricia O'Shea:** conceptualisation, writing – original draft, writing – review and editing, supervision. **Hannah Healy:** conceptualisation, writing – original draft, methodology, writing – review and editing, resources. **Anne MacFarlane:** conceptualisation, writing – review and editing, supervision.

## Funding

The authors have nothing to report.

## Conflicts of Interest

The authors declare no conflicts of interest.

## Patient or Public Contribution

People with lived experience were involved at all stages of the book project. A co‐editor living with chronic pain shaped the original concept, governance and ethical direction of the book, ensuring lived expertise was positioned equally alongside professional knowledge. A total of 11 contributors with lived experience co‐authored chapters for the book, actively shaping content, language and representation. Although not all are authors in this viewpoint, in preparing this reflection contributors to the book were invited to review and comment on draft themes and interpretations, and all were asked to consent to publication. Their feedback informed the analysis and ensured alignment with Participatory Learning and Action (PLA) principles, particularly in relation to power‐sharing and authorship.

## Data Availability

The data that support the findings of this study are available on request from the corresponding author. The data are not publicly available due to privacy or ethical restrictions.
